# Notes from the Field: Fatal Pneumonic Tularemia Associated with Dog Exposure — Arizona, June 2016

**DOI:** 10.15585/mmwr.mm6633a5

**Published:** 2017-08-25

**Authors:** Hayley Yaglom, Edwin Rodriguez, Marlene Gaither, Mare Schumacher, Natalie Kwit, Christina Nelson, Joel Terriquez, Jacob Vinocur, Dawn Birdsell, David M Wagner, Jeannine Petersen, Kiersten Kugeler

**Affiliations:** ^1^Arizona Department of Health Services; ^2^Coconino County Public Health Services District, Arizona; ^3^Division of Vector-Borne Diseases, National Center for Emerging and Zoonotic Infectious Diseases, CDC; ^4^Northern Arizona Healthcare; ^5^Northern Arizona University.

On June 15, 2016, Arizona public health officials were notified of a presumptive positive *Francisella tularensis* blood culture result from a woman aged 73 years with pulmonary sarcoidosis who had recently died from respiratory failure. She had been taking amoxicillin for a dental infection. She was evaluated on June 6 for 4 days of fever, myalgia, anorexia, and diarrhea. Because of suspected colitis she was advised to discontinue amoxicillin; she declined hospital admission. Two days later, she was hospitalized for shortness of breath and confusion. Chest radiography revealed a right lower lobe pneumonia and an effusion. This was treated with cefepime and intravenous doxycycline. On June 8, her stool tested positive for *Clostridium difficile* toxin A/B by polymerase chain reaction, requiring treatment with metronidazole and vancomycin. Her condition deteriorated, and she died on June 11. Tularemia was not suspected as cause of illness until June 17 when a blood culture collected on June 6 was confirmed as *F. tularensis*, a Tier 1 select agent; no laboratory exposures occurred.

The patient lived in a semirural area of northern Arizona, did not engage in outdoor activities, and had no known history of insect bites, exposure to animal carcasses or untreated water. She traveled to Hawaii May 16–26, returning approximately 11 days before illness onset. Postmortem exam revealed no bites, abscesses, or lymphadenopathy.

The patient’s dog was noted to be lethargic and anorexic in late May, 3 days after being found with a rabbit carcass in its mouth. The patient and dog had frequent close contact. Serum from the dog, obtained approximately 3 weeks after its illness and the patient’s death, had a *F. tularensis*-specific titer of 1:256. An assessment of the property on June 23 revealed numerous rabbits and one squirrel carcass with *F*. *tularensis* DNA detected in its liver and spleen. Genotyping of *F. tularensis* from squirrel and human samples showed both infections were attributable to an A.II strain.

Approximately 125 human tularemia cases are reported in the United States annually. Humans are infected through arthropod bites, contact with infectious tissues, inhalation, or ingestion (*1*). Symptoms commonly begin 3–5 days after exposure and can include fever, skin lesions, lymphadenopathy, difficulty breathing, and diarrhea (*1*).

Two *F. tularensis* subspecies, *tularensis* (Type A) and *holarctica* (Type B), cause human tularemia (*1*,*2*). Distinct clades within Type A (A.I and A.II) are associated with different virulence in humans and laboratory animals (*2*,*3*). A.II strains are localized to the western United States and associated with milder illness than are A.I strains (*2*,*3*).

Based on the patient’s respiratory symptoms, radiographic findings, and lack of alternative exposure history, exposure likely occurred at her property through inhalation of *F. tularensis*, potentially via close contact with her dog. The dog might have transmitted infectious material through oral secretions after mouthing an infected carcass or brought infectious material on its fur into contact with the patient. Human illness linked to dogs has been documented (*3*).

The role of pulmonary sarcoidosis in this patient’s illness is unclear, but it might have contributed to the severe outcome of infection with an A.II strain (*4*). Concurrent infection with *C. difficile* might also have exacerbated the clinical course of tularemia. Diagnosis of tularemia is challenging because symptoms are nonspecific and exposure history is often unclear. Thorough ascertainment of animal exposures, including nature of contact, might refine clinical suspicion for specific zoonoses. Preventing exposure and implementing early, appropriate therapy can reduce morbidity and mortality. Additional information is available at https://www.cdc.gov/tularemia.
